# Association between complete revascularization of the coronary artery and clinical outcomes in peripheral artery disease: a sub-analysis of the I-PAD Nagano registry

**DOI:** 10.1007/s00380-023-02251-y

**Published:** 2023-04-13

**Authors:** Tamon Kato, Yasushi Ueki, Masatoshi Minamisawa, Takashi Miura, Yushi Oyama, Naoto Hashizume, Daisuke Yokota, Minami Taki, Keisuke Senda, Yoshiteru Okina, Tadamasa Wakabayashi, Koki Fujimori, Kenichi Karube, Takahiro Sakai, Hidetomo Nomi, Hisanori Yui, Yusuke Kanzaki, Keisuke Machida, Shusaku Maruyama, Ayumu Nagae, Tatsuya Saigusa, Soichiro Ebisawa, Ayako Okada, Hirohiko Motoki, Koichiro Kuwahara, the I-PAD investigators

**Affiliations:** 1 Department of Cardiovascular Medicine, Shinshu University School of Medicine, 3-1-1 Asahi, Matsumoto, Nagano 390-8621 Japan; 2 Department of Cardiology, Nagano Municipal Hospital, Nagano, Japan; 3 Department of Cardiology, Shinonoi General Hospital, Nagano, Japan; 4 Department of Cardiology, Nagano Red-Cross Hospital, Nagano, Japan; 5 Department of Cardiology, Iida Hospital, Nagano, Japan; 6 Department of Cardiology, Saku General Hospital, Nagano, Japan; 7 Department of Cardiology, Aizawa Hospital, Nagano, Japan; 8 Department of Cardiology, Joetsu General Hospital, Niigata, Japan; 9 Department of Cardiology, Suwa Central Hospital, Nagano, Japan; 10 Department of Cardiology, Suwa Red-Cross Hospital, Nagano, Japan; 11 Department of Cardiology, Okaya City Hospital, Nagano, Japan; 12 Department of Cardiology, Ina Central Hospital, Nagano, Japan

**Keywords:** Complete revascularization, Peripheral artery disease, Coronary artery disease, Major adverse cardiovascular events, Endovascular therapy

## Abstract

Peripheral artery disease (PAD) is commonly caused by atherosclerosis and has an unfavorable prognosis. Complete revascularization (CR) of the coronary artery reduces the risk of major adverse cardiovascular event (MACE) in patients with coronary artery disease (CAD). However, the impact of CR in patients with PAD has not been established to date. Therefore, we evaluated the impact of CR of CAD on the five-year clinical outcomes in patients with PAD. This study was based on a prospective, multicenter, observational registry in Japan. We enrolled 366 patients with PAD undergoing endovascular treatment. The primary endpoint was MACE, defined as a composite of all-cause death, non-fatal myocardial infarction, and non-fatal stroke. After excluding ineligible patients, 96 and 68 patients received complete revascularization of the coronary artery (CR group) and incomplete revascularization of the coronary artery (ICR group), respectively. Freedom from MACE in the CR group was significantly higher than in the ICR group at 5 years (66.7% vs 46.0%, *p* < 0.01). Multivariate analysis revealed that CR emerged as an independent predictor of MACE (Hazard ratio: 0.56, 95% confidential interval: 0.34–0.94, *p* = 0.03). CR of CAD was significantly associated with improved clinical outcomes in patients with PAD undergoing endovascular treatment.

## Introduction

Peripheral artery disease (PAD) is commonly caused by atherosclerosis. A number of previous studies have reported an increased risk of mortality, cardiovascular mortality, and ischemic evnets such as myocardial infarction and stroke in patients with lower extremity artery disease (LEAD) [1, 2]. In patients undergoing surgery for LEAD, the probability of significant concomitant coronary artery disease (CAD) on coronary angiography is 50–60% [3–5]. In the Reduction of Atherothrombosis for Continued Health registry, polyvascular disease was associated with a worse prognosis [6, 7], and 57% of the patients with LEAD had CAD [6]. Previous observational studies and meta-analysis have reported that complete revascularization (CR) reduces adverse events in patients with CAD [8–10]. CR is especially important in the high-risk patient subsets such as acute coronary syndrome and the elderly patients [11, 12]. To date, the clinical benefit of CR of the coronary artery on clinical outcomes in patients with PAD has not been well established. Therefore, we aimed to evaluate the impact of CR of CAD on clinical outcomes among patients with PAD undergoing endovascular treatment (EVT) for lower limb arteries.

## Methods

### Study design and patient population

The Improving Prognosis of Peripheral Artery Disease patients undergoing EVT in Nagano, Japan (I-PAD Nagano) registry was a prospective, multicenter, observational registry designed to provide up to 60 months of clinical follow-up. A total of 366 patients undergoing EVT for symptomatic PAD were enrolled at 11 institutions between August 2015 and August 2016. For the current subanalysis, patients not undergoing coronary angiography or those without significant coronary artery stenosis were excluded. Patients were divided into the following two groups: the CR group and incomplete revascularization (ICR) group. The I-PAD registry was approved by the medical ethics committee of the Shinshu University School of Medicine (No. 3187). The study and procedures were conducted in accordance with the Declaration of Helsinki. A written informed consent was taken from the enrolled patients. The study was registered with the University Hospital Medical Information Network Clinical Trials Registry, as accepted by the International Committee of Medical Journal Editors (No. UMIN000018297).

### Definitions

CAD was defined as ≥ 50% stenosis of a coronary artery on angiography, or a history of coronary artery bypass graft surgery, PCI, or myocardial infarction. CR was defined as residual Syntax score = 0 [13]. The SYNTAX score is the sum of the points assigned to each individual lesion identified in the coronary tree with > 50% diameter narrowing in vessels > 1.5-mm diameter. Each segment is given a score of 1 or 2 based on the presence of disease. Subsequently, this score is weighted based on a chart, with values ranging from 3.5 for the proximal left anterior descending artery to 5.0 for left main, and 0.5 for smaller branches [14]. Cerebrovascular disease was defined according to the hospital or a reported diagnosis of transient ischemic attack or ischemic stroke by a neurologist. Heart failure was defined as a previous diagnosis of heart failure, history of hospitalization for heart failure, or current treatment for heart failure. Diabetes mellitus was defined as a hemoglobin A1c level of ≥ 6.5%, random plasma glucose ≥ 200 mg/dL or treatment with oral hypoglycemic agents or insulin injection. Hypertension was defined as a systolic blood pressure ≥ 140 mmHg, diastolic blood pressure ≥ 90 mmHg, or ongoing therapy for hypertension. Dyslipidemia was defined as a serum cholesterol concentration of ≥ 220 mg/dL, a low-density lipoprotein cholesterol concentration of ≥ 140 mg/dL, or current treatment with lipid lowering agents. Chronic limb-threatening ischemia (CLTI) was defined as a patient with objectively documented PAD and any of the clinical symptoms or signs of ischemic rest pain with confirmatory hemodynamic studies or gangrene involving any portion of the lower limb or foot, except for pure venous ulcers, pure traumatic wounds, acute limb ischemia (symptoms present for two weeks or less), embolic disease, and nonatherosclerotic chronic vascular conditions of the lower extremity (e.g., vasculitis, Buerger disease, radiation arteritis). Body mass index was defined as weight in kilograms divided by the square of the patient’s height in meters. Estimated glomerular filtration (eGFR) was calculated with the following formula: male: eGFR (mL/min/1.73m^2^) = 194 × creatinine (Cr)^−1.094^ × age^−0.287^, female: eGFR = 194 × Cr^−1.094^ × age^−0.287^ × 0.739.

### Study endpoint

Primary endpoint was major adverse cardiovascular events (MACE), defined as a composite of all-cause death, myocardial infarction, and stroke at five years. Secondary endpoints were survival and major adverse cardiovascular and leg events (MACLE), defined as a composite of all-cause death, myocardial infarction, stroke, major amputation, reintervention for PAD. Myocardial infarction (MI) was defined according to the 3rd universal definition of MI [15]. Target extremity revascularization included either EVT or a surgical procedure [16]. All above-ankle amputations were considered a major amputation [17].

### Statistical analysis

Continuous variables were reported as median (interquartile range) and compared by using Wilcoxon-Mann–Whitney *U* tests, as appropriate. Categorical data were calculated as frequencies (percentages) and were compared with the chi-square test. Survival curves were constructed for time-event variables with Kaplan–Meier estimates and compared using the log-rank test. Cox regression analysis was performed to test the prognostic significance of complete revascularization for MACE. CR was adjusted by clinically important variables reported by previous studies [18–22]. A *P* value < 0.05 was considered statistically significant in all analyses. Data were analyzed using JMP 11 (SAS Institute, Cary, NC).

## Results

### Patient characteristics

**Fig. 1:**
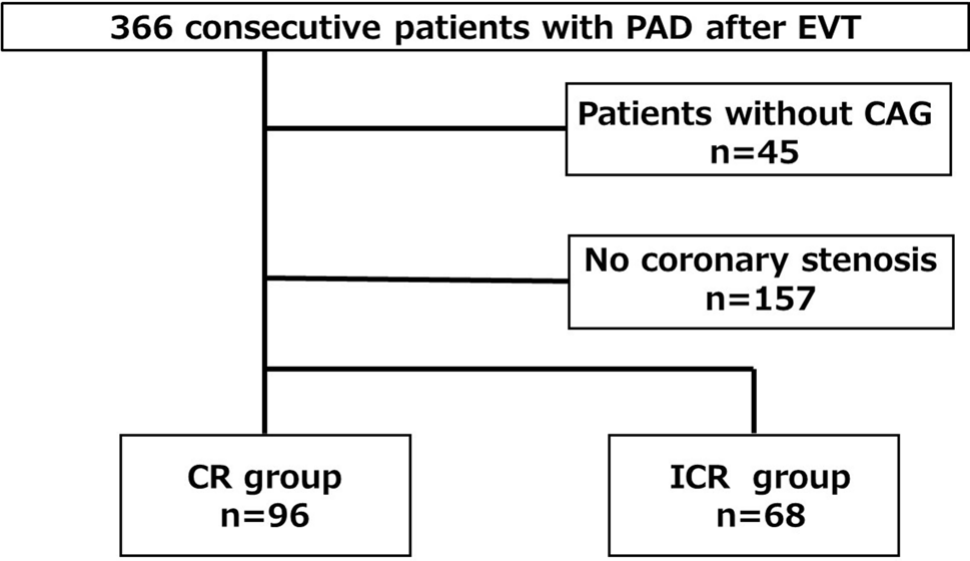
Patient flow chart. *CAG* coronary angiography, *PAD* peripheral artery disease, *EVT* endovascular treatment

Of 366 patients enrolled in the I-PAD Nagano registry, those who did not undergo coronary angiography (*n* = 45) or those who did not have significant coronary artery stenosis (*n* = 157) were excluded. As a result, 164 patients were analyzed for the current study (CR group: *n* = 96, ICR group: *n* = 68) (Fig. 1). In the CR group, CR with PCI was performed within 6 months after EVT. Baseline characteristics are summarized in Tables 1 and 2. There were no significant differences in baseline characteristics between groups. A median residual Syntax score in the ICR group was 6.

**Table 1:** Baseline characteristics

Variables	Complete revascularization (*n* = 96)	Incomplete revascularization (*n* = 68)	*P* value
Age (years)	73 [67, 80]	75 [68, 81]	0.30
Male	70 (72.9)	55 (80.9)	0.24
Body mass index (kg/m^2^)	22.5 [20.1, 25.4]	22.1 [19.9, 24.6]	0.49
Hypertension	86 (89.6)	57 (83.8)	0.28
Dyslipidemia	63 (65.6)	41 (60.3)	0.49
Diabetes mellitus	62 (64.6)	45 (66.2)	0.83
Smoking	54 (56.3)	46 (67.6)	0.12
CVD	17 (17.7)	19 (27.9)	0.12
Myocardial infarction	24 (25.0)	27 (39.7)	0.05
Heart failure	13 (13.5)	17 (25.0)	0.06
Hemodialysis	25 (26.0)	23 (33.8)	0.28
Rutherford classification			0.05
I	2 (2.1)	4 (5.9)	
II	11 (11.5)	9 (13.2)	
III	51 (53.1)	27 (39.7)	
IV	19 (19.8)	7 (10.3)	
V	11 (11.5)	16 (23.5)	
VI	2 (2.1)	5 (7.4)	
CLTI	32 (30.2)	28 (38.2)	0.30
LVEF (%)	65.3 [58.2, 69.5]	61.5 [50.3, 69.1]	0.17
Albumin (g/mL)	3.9 [3.6, 4.2]	3.8 [3.5, 4.2]	0.34
Hemoglobin (g/dL)	13.2 [11.5, 14.6]	12.4 [11.4, 13.9]	0.16
HbA1c (%)	6.7 [5.9, 7.2]	6.4 [5.8, 7.1]	0.22
eGFR (mL/min/1.73 m^2^)	54.1 [12.4, 65.4]	41.1 [14.2, 59.5]	0.20
CRP (mg/dL)	0.21 [0.05, 0.56]	0.20 [0.08, 0.80]	0.34
BNP (pg/mL)	102.6 [46.8, 249.8]	133.5 [58.7, 320.0]	0.26
Total Cholesterol (mg/dL)	171 [143.8, 199]	170 [151, 196]	0.35
LDL-C (mg/dL)	91 [73.8, 115.3]	99 [80, 117]	0.21
HDL-C (mg/dL)	48 [42, 60]	49 [41, 61]	0.65
TG (mg/dL)	104 [73, 161]	104 [76, 158]	0.78

Data are shown as median [interquartile range], or n (percentage)

*CVD* cerebrovascular disease, *CLTI* chronic limb threatening ischemia, *LVEF* left ventricular ejection fraction, *ABI* ankle–brachial index, *eGFR* estimated glomerular filtration rate, *CRP* C-reactive protein, *BNP* brain natriuretic peptide, *LDL-C* low density lipoprotein cholesterol, *HDL-C* high density lipoprotein cholesterol, *TG* triglyceride

**Table 2:** Lesion characteristics

Variables	Complete revascularization (*n* = 96)	Incomplete revascularization (*n* = 68)	*P* value
Target vessel			0.48
Iliac	35 (36.5)	26 (38.2)	
Femoropopliteal	50 (52.1)	38 (55.9)	
BTK	11 (11.5)	4 (5.9)	
TASC classification			0.51
A	24 (25.0)	20 (29.4)	
B	20 (20.8)	20 (29.4)	
C	14 (14.6)	9 (13.2)	
D	30 (31.3)	16 (23.5)	
CTO of peripheral artery	35 (36.5)	26 (38.2)	0.82

Data are shown as *n* (percentage)

*BTK* below the knee, *TASC* Trans-Atlantic Inter-Society Consensus, *CTO* chronic total occlusion

### Clinical outcomes

**Fig. 2:**
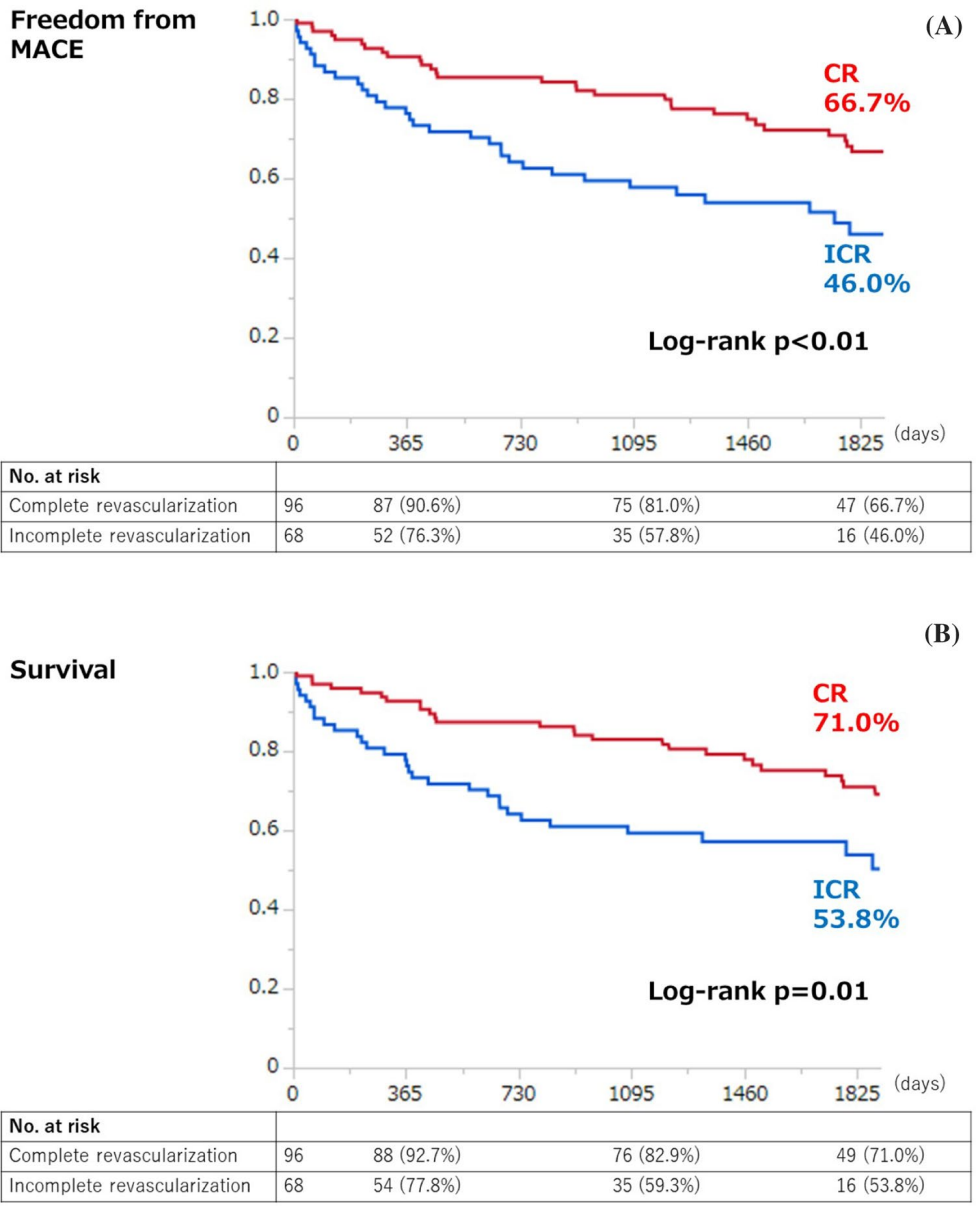

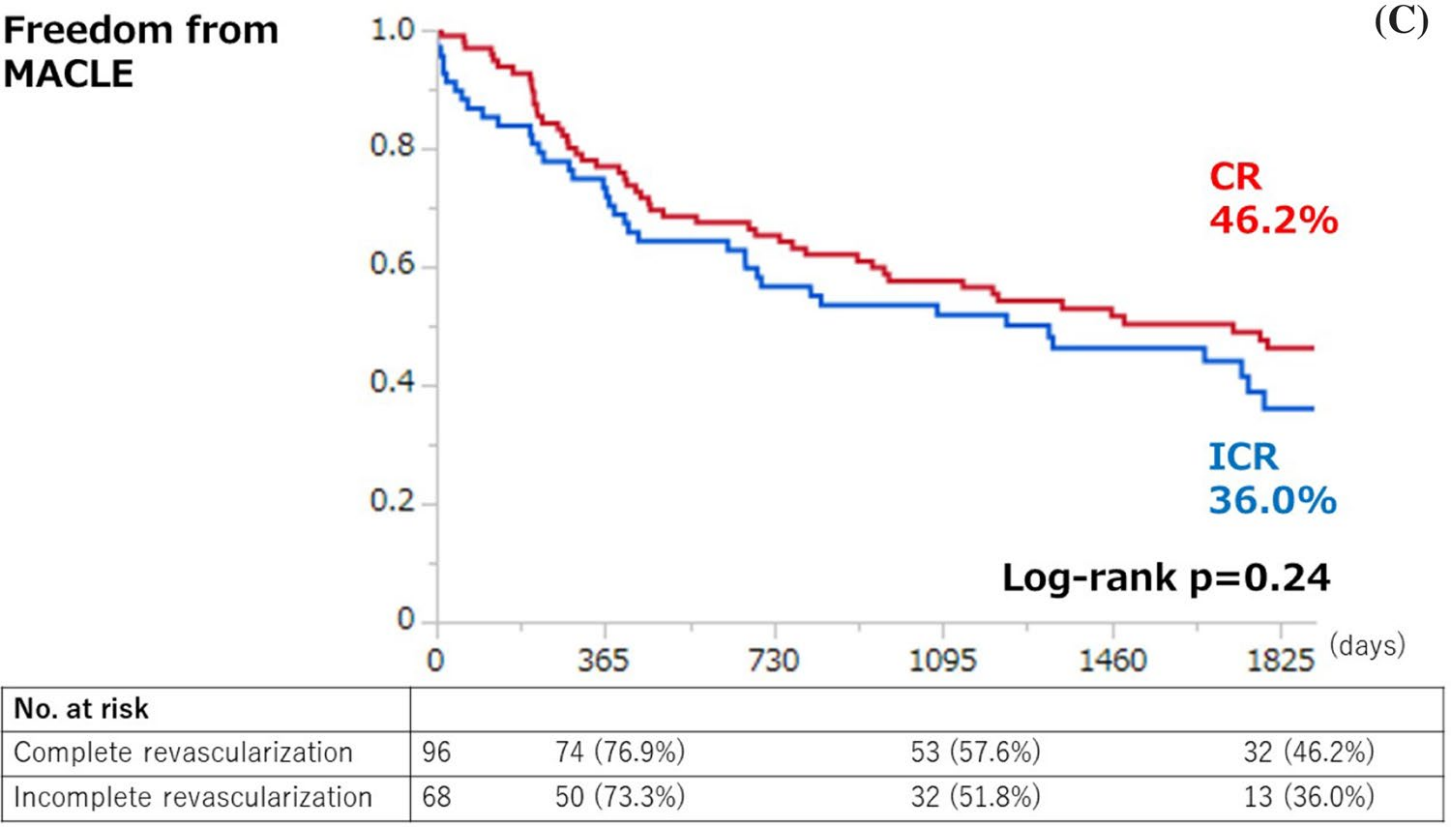
Kaplan–Meier curves for freedom from MACE (**A**), survival (**B**), and freedom from MACLE (**C**) at five years. *MACE* major adverse cardiovascular events, *MACLE* major adverse cardiovascular limb events, *CR* complete vascularization of the coronary artery, *ICR* incomplete revascularization of the coronary artery

**Fig. 3:**
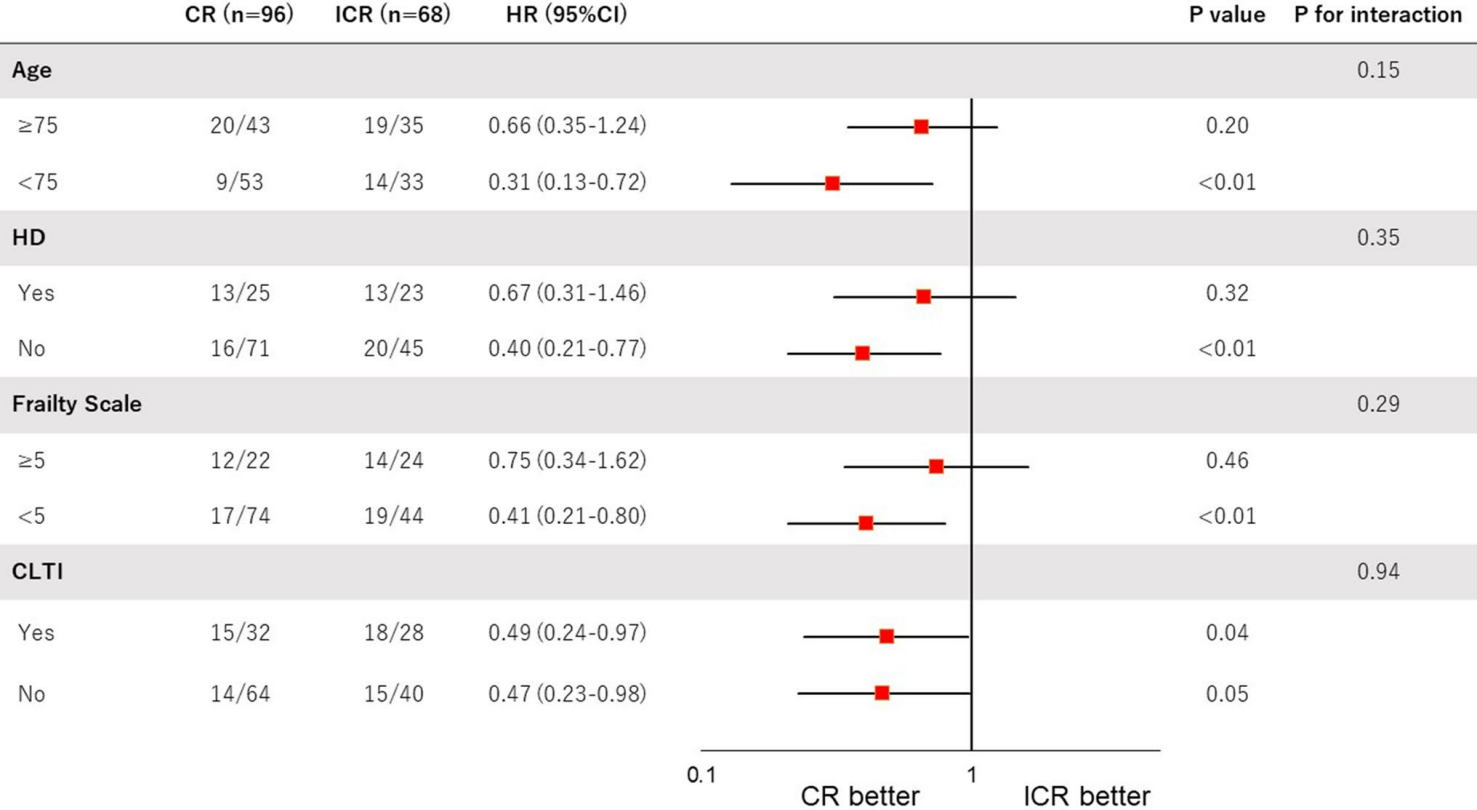
Stratified analyses of MACE at 5 years across major subgroups. *CR* complete revascularization of the coronary artery, *ICR* incomplete revascularization of the coronary artery, *BMI* body mass index, *CLTI* chronic limb-threatening ischemia

**Fig. 4:**
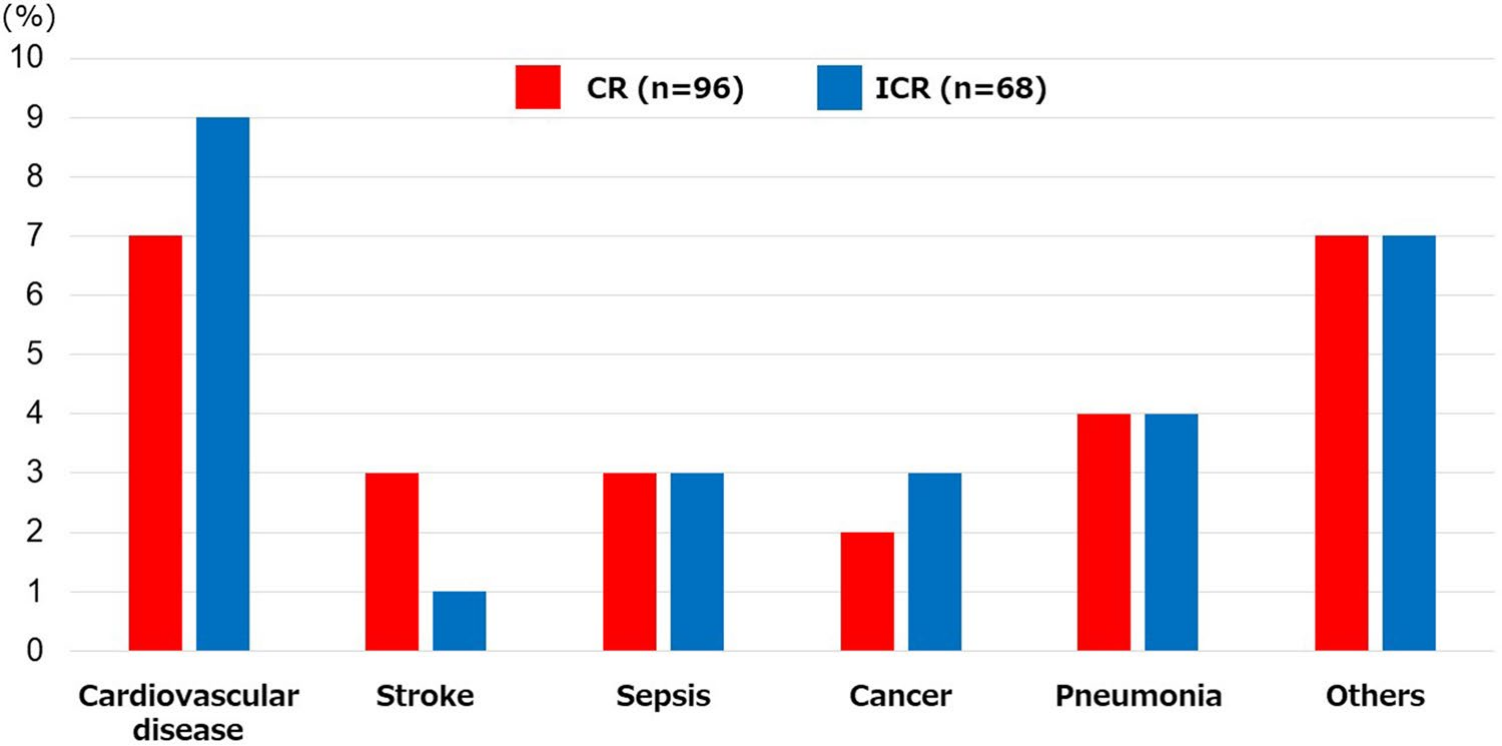
Causes of death. *CR* complete revascularization of the coronary artery, *ICR* incomplete revascularization of the coronary artery

Kaplan–Meier analysis for clinical outcomes were summarized in Fig. 2. The freedom from MACE was significantly higher in the CR group compared with the ICR group (66.7% vs. 46.0%, *p* < 0.01) (Fig. 2A). The survival was significantly higher in the CR group than in the ICR group (71.0% vs. 53.8%, *p* = 0.01) (Fig. 2B). There was no significant difference in MACLE (46.2% vs. 36.0%, respectively, *p* = 0.24) (Fig. 2C). CR was significantly associated with a lower risk of MACE among patients < 75 years (hazard ratio [HR] 0.31, 95% confidence interval [CI] 0.13–0.72), those without hemodialysis (HR 0.40, 95%CI 0.21–0.77), those with low frailty scale (i.e. < 5) (HR 0.41, 95%CI 0.21–0.80), and those with CLTI (HR 0.49, 95%CI 0.24–0.97), whereas no significant interaction was observed across patient subsets in a stratified analysis (Fig. 3). There were 55 deaths in the current study (CR 28 vs ICR 27); the major cause of death was cardiac death in both groups (CR 7.3% vs. ICR 13.2%) (Fig. 4).

### Multivariable COX analysis

The results of multivariable Cox regression analysis for MACE are shown in Table 3. CR of the coronary artery (HR 0.56, 95%CI 0.34–0.94, *p* = 0.03), age (HR 1.04, 95%CI 1.01–1.08, *p* < 0.01), body mass index (HR 0.87, 95%CI 0.80–0.94, *p* < 0.01), hemodialysis (HR 2.63, 95%CI 1.41–4.72, *p* < 0.01), CLTI (HR 2.29, 95%CI 1.36–3.85, *p* < 0.01), and heart failure (HR: 2.71, 95% CI: 1.51–4.84, *p* < 0.01) were significantly associated with MACE.

**Table 3:** COX multivariate analysis for MACE at 5 years

Risk factor	Hazard ratio	95% confidence interval	*P* value
Age (years)	1.04	1.01–1.08	< 0.01
Body mass index (kg/m^2^)	0.87	0.80–0.94	< 0.01
Hemodialysis	2.63	1.41–4.72	< 0.01
CLTI	2.29	1.36–3.85	< 0.01
Heart failure	2.71	1.51–4.84	< 0.01
CR in coronary artery	0.56	0.34–0.94	0.03

*MACE* major adverse cardiovascular event, *CLTI* chronic limb threatening ischemia, *CR* complete revascularization

## Discussion

The I-PAD Nagano registry is a prospective, multicenter observational study designed to evaluate the clinical outcomes in PAD patients undergoing EVT. Major findings of the current study are as follows: (1) the freedom from MACE and survival were significantly higher in the CR group; (2) there was no significant differene in MACLE between groups; and (3) cardiovascular death was the most common cause of death in both groups.

CAD and PAD are common manifestations of systemic atherosclerosis and both diseases share several common risk factors. A number of previous studies have demonstrated clinical benefits of CR in patients with CAD. A prior meta-analysis reported that CR reduced adverse events in patients with CAD [8]. The impact of CR is reportedly more relevant in elderly patients and those with acute coronary syndrome [12, 23]. In the latest practice guidelines, there are no specific recommendations regarding CR of CAD in patients with PAD [24]. This is the first study to evaluate the clinical relevance of CR in patients with PAD. Given an increased risk of ischemic events in patients with PAD, the findings of the current study suggest the potential benefit of CR of the coronary arteries in this population.

In the current study, CR was performed in 58.5% (*n* = 96/164) of patients, which was similar to the previous studies (31–61%) [9]. In our cohort, the patients in the ICR group were numerically older than those in the CR group. Elderly patients tend to have more coexisting comorbidities including renal dysfunciton and heart failure, which makes CR more challenging.

We performed subgroup analyses according to age, dialysis, frailty, and CLTI. Although CR was significantly associated with a lower risk of MACE among younger patients, those without hemodialysis, those with low frailty scale, and those with CLTI, no significant interaction was observed. Previous studies have reported that CR was effective in the elderly patients with CAD [11, 12]. Since PAD is an advanced stage of systemic arteriosclerosis, it might be important to perform CR at an earlier stage to avoid MACE in patients with PAD.

In the current study, there were 55 deaths within a 5-year observation period (CR: 28 cases [29.2%] vs. ICR: 27 cases [39.7%], *p* = 0.11). Cardiac death was the most frequent cause of death in both groups. There was no significant difference between the CR and ICR groups. Seven (7.3%) and nine (13.2%) cases of cardiac death were observed in the CR and ICR group, respectively, during the 5-year observational period (*p* = 0.09). In the current study, CR was not strongly associated with an increased mortality. The second and third major causes of death were pneumonia and sepsis, respectively, in both groups.

## Limitations

There are several limitations to the current study. First, this was a non-randomized trial with small number of patients. Second, although multivariable adjustments were performed for the current study, there may be several potential unmeasured confounding factors inherent to observational data. Third, coronary angiography and PCI were peformed at the discretion of physicians. Systematic assessments of myocardial ischemia with fractional flow reserve or non-invasive imaging were lacking in the current study, which may impact the findings of the current study. Fourth, the reasons for not achieving CR were not available. Fifth, Syntax score at baseline and the details in PCI were not available in the current study and therefore they were not incorporated into the multivariate analysis.

In conclusion, CR of the coronary artery was significantly associated with freedom from MACE and survival in patients with PAD in comparison to ICR. Whether CR improves outcomes of PAD patients requires further investigation.

## Data Availability

The datasets generated during and/or analysed durning the current study are available from the corresponding author on reasonable request.
